# Thank you reviewers- CytoJournal 2007

**DOI:** 10.1186/1742-6413-4-4

**Published:** 2007-01-30

**Authors:** Vinod B Shidham, Barbara F Atkinson

**Affiliations:** 1 Executive editor and coeditor-in-chief, CytoJournal, Dept of Pathology, Medical College of Wisconsin, Milwaukee, WI, USA; 2 Coeditor-in-chief, CytoJournal, University of Kansas School of Medicine, Kansas City, KS, USA

## Abstract

Significant efforts, time, and resources are devoted for peer-reviewing numerous CytoJournal manuscripts. The Editorial Board of CytoJournal shares a significant proportion of this activity. Additional peers are requested to join periodically as 'academic editors' and reviewers to review CytoJournal manuscripts. We thank all the reviewers and academic editors for their time and efforts for completing the peer-review of CytoJournal manuscripts during 2006. The continued success of this important academic exercise depends on their continued enthusiasm to support with their highest standards. We also thank all the contributing authors for selecting CytoJournal and supporting open access initiative, which allows retention of the copyrights to their corresponding academic accomplishments.

## Editorial

In accordance with the 'close review' policy of CytoJournal, the peer-reviewers are not identified during the review process or in the published articles. CytoJournal periodically designates an 'academic editor' to some manuscripts for various reasons including specialized nature of topic to conflict of interest for conducting and completing the peer-review process for reviewing various manuscripts submitted to CytoJournal. The academic editors are acknowledged in the corresponding articles [1]. As a token of appreciation and thanks, CytoJournal periodically publishes the list of all the academic editors and reviewers involved in the peer-reviewing process of all the manuscripts submitted to CytoJournal irrespective of their publication status [2-25].

On behalf of CytoJournal editorial board and Cytopathology-Foundation Inc, we thank all reviewers and academic editors participating in CytoJournal peer-review process during 2006 (Table 1). As some of the submitted manuscripts are not accepted for publication, the academic editors reviewing such manuscripts are thanked collectively in this article. The quality of peer-review is extremely important to continue and enhance the high standards of CytoJournal for publishing the best work [26]. Their time and efforts for reviewing the manuscripts to improve their quality is commendable. A high standard of peer-review process in timely fashion is very important component of CytoJournal [27].

**Table 1:** CytoJournal reviewers and academic editors* (2007).

Akobeng	Anthony	Krishnamurthy	Savitri
Almagro	Urias	Kroft	Steven
Al-Quran	Samer	Kurtycz	Daniel
Ashfaq	Raheela	Lin	Fan
Bentz	Brandon	Logani	Sanjay
Bose	Shikha	Meisels	Alexander
Brachtel	Elena	Markelova	Natalia
Bubendorf	Lukas	Masood	Shahla
Caraway	Nancy	Müller	Henning
Chang	Chung-Che (Jeff)	Niamtu	Joseph
Chhieng	David	Novoa-Takara	Louis
Clark	Douglas	O'Malley	Dennis
Cochand-Priollet	Beatrix	Pasieka	J
Coleman	Dulcie	Powers	Celeste N
Crabtree	William	Pollard	Tom Joseph
Das	Dilip K.	Rao	Nagarjun
Davey	Diane	Rimm	David
Dejmek	Annika	Ross	Try
DeMay	Richard	Schneider	Volker
Domanski	H	Shidham	Vinod B.
Drew	Peter	Sidawy	Mary
Dunn	Bruce	Siddiqui	Momin
Ehya	Hormoz	Singleton	Timothy
Eltoum	Isam	Skoog	Lambert
Erozan	Yener	Sneige	Nour
Field	Andrew Stanley	Stanley	Michael
Frable	William	Stanley	Michael
Fukunaga	M	Stelow	Edward
Gal	AA	Stoler	Mark
Hoda	Rana	Orell	Svante R.
Ibrahim	Sherif	Tani	Edneia
Jensen	David	Wang-Rodriguez	Jessica
Jhala	Nirag	Verma	Kusum
Herbert	Amanda	Wilbur	David
Kapila	Kusum	Wilson	Carla
Katz	Ruth Louise	Ylagan	Lourdes
Kilpatrick	Scott	Young	Nancy

*All peer-reviewers and academic editors participating in reviewing CytoJournal manuscripts during 2006. We apologize in advance for any omissions due to oversight. Please contact by email (see the title page) and communicate the lapse, we will include the missing reviewer in the next list. We thank you in advance for your understanding.

We also thank all the contributing authors for supporting the 'open access' initiative of CytoJournal. 'Open access' extends numerous benefits to the authors including the retention of their copyrights of their published material [28].

We invite all the experts in cytopathology and related areas to join the ever increasing demand of good reviewers by sending e-mail to cytojournal@mcw.edu along with recent curriculum vitae.

Sincerely,

Vinod B. Shidham, MD, FRCPath, FIAC

Executive editor and co-editor-in-chief

Barbara F. Atkinson, MD

Co-editor-in-chief
